# A pipeline for senolytics

**DOI:** 10.1172/JCI180558

**Published:** 2024-05-01

**Authors:** Sundeep Khosla

**Affiliations:** Division of Endocrinology and Kogod Center on Aging, Mayo Clinic College of Medicine, Rochester, Minnesota, USA.

## Abstract

There is intense interest in identifying compounds that selectively kill senescent cells, termed senolytics, for ameliorating age-related comorbidities. However, screening for senolytic compounds currently relies on primary cells or cell lines where senescence is induced in vitro. Given the complexity of senescent cells across tissues and diseases, this approach may not target the senescent cells that develop under specific conditions in vivo. In this issue of the *JCI*, Lee et al. describe a pipeline for high-throughput drug screening of senolytic compounds where senescence was induced in vivo and identify the HSP90 inhibitor XL888 as a candidate senolytic to treat idiopathic pulmonary fibrosis.

## The complexity of cellular senescence

With population aging, considerable resources are being invested in developing pharmaceutical interventions to increase healthspan and perhaps even lifespan. Underpinning this effort is the geroscience hypothesis, which posits that manipulation of fundamental aging mechanisms will delay, in parallel, the appearance or severity of multiple chronic diseases because these diseases share the same underlying risk factor—aging (1). In the most current iteration, 12 fundamental aging mechanisms have been defined (2). Of these, cellular senescence has received particular attention in recent years following the observations that reducing the burden of senescent cells in aged mice, using either genetic (3) or pharmacologic (4) approaches, increased healthspan and lifespan.

Although the definition continues to evolve, senescent cells are currently defined by the following key characteristics (5): upregulation of the cyclin-dependent kinase inhibitors, *p16^Ink4a^* and/or *p21^Cip1^*, growth arrest, evidence of DNA damage, upregulation of antiapoptotic pathways, and the senescence-associated secretory phenotype (SASP), which consists of cytokines, proteases, bioactive lipids, and other proinflammatory molecules (6). The SASP is of particular importance, as it explains why only a minority of senescent cells in aged tissues (approximately 10%) can cause local as well as systemic tissue damage or dysfunction (7). Conversely, reducing the SASP either through killing senescent cells using senolytics (4) or inhibiting the secretion of the SASP (e.g., using JAK inhibitors (8)) has now been shown to ameliorate a number of aging phenotypes, at least in mice.

Although cellular senescence is unquestionably a hallmark of aging, it is clear that cells expressing features of senescence (e.g., increased *p16^Ink4a^* and/or *p21^Cip1^* expression and a highly inflammatory secretome) also appear following tissue injury in the skin (9), bone (10), muscle (11), and lung (12). In contrast to the persistence of senescent cells with aging, these injury-related senescent cells are generally cleared as the tissue heals (10). Moreover, although initially thought to facilitate tissue repair (9), it appears that these injury-related senescent cells may either facilitate (9) or impair (10, 11) tissue healing, depending on the tissue and type of injury. In addition, possible similarities or differences between the classical age-associated versus injury-related senescent cells remain to be clearly defined. Finally, individual diseases, such as idiopathic pulmonary fibrosis, diabetes, osteoporosis, dementia, and others may be associated with specific types of senescent cells and a SASP unique to that particular disease process (13). But it does not end there, as, even with aging, the characteristics and the SASP of senescent cells across tissues appears to be quite different (14).

This enormous complexity of senescent cell phenotypes with aging, injury, or disease challenges the fundamental tenet of the geroscience hypothesis. Specifically, whether it is even possible to develop broad-spectrum senolytics that target a fundamental aging mechanism that can clear senescent cells across tissues. Whether this could occur with aging as well as in various diseases, or if we are going to need to develop relatively tissue- or disease-specific senolytics is another element of the question. Although the answer to this question remains unresolved, to the extent that the latter is the case, the study by Lee and colleagues (15) in the current issue of the *JCI* is particularly relevant.

## A pipeline for screening senolytic compounds

Lee and colleagues (15) address the key problem that screening for senolytic compounds, as is currently done, relies on primary cells or cell lines where senescence is induced in vitro. Given the complexity of senescent cells across the tissues and diseases noted above, this approach may not target the senescent cells that develop under specific conditions in vivo. To address this issue, the authors took advantage of a model, previously developed by the same laboratory, using INKBRITE mice (12), where the endogenous *p16^Ink4a^* promoter drives three copies of GFP, resulting in highly sensitive *p16^Ink4a^-*reporter mice. This strategy circumvented the longstanding problem that *p16*^Ink4a+^ cells can be difficult to isolate due to the relatively low expression of *p16^Ink4a^*, even in senescent cells.

**Figure 1:**
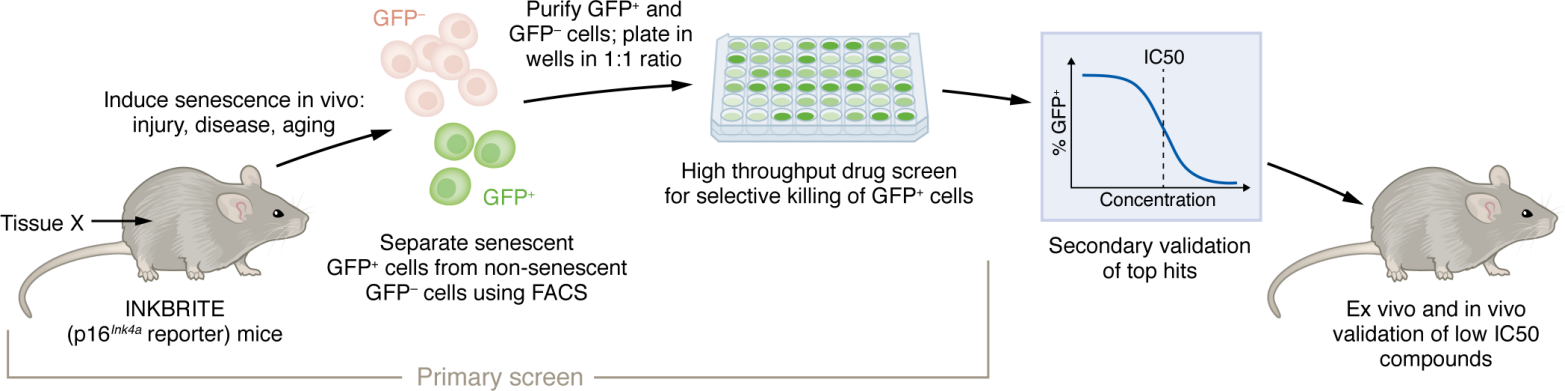
Lee et al. developed an approach for high-throughput screening of senolytic compounds. In Lee et al. (15), senescence is induced in mice that express a highly sensitive *p16^Ink4a^* reporter, termed INKBRITE, followed by fluorescence-activated cell sorting (FACS) for GFP^+^ (*p16*^Ink4a+^) and GFP^–^ (*p16*^Ink4a–^) cells. The purified GFP^+^ and GFP^–^ cells are then mixed in a 1:1 ratio and plated into multi-well plates for high-throughput screening of candidate senolytic compounds, which are selected based on selective killing of GFP^+^ cells (GFP^+^-to-GFP^–^ cell ratio below a specific threshold). These compounds are then carried forward into secondary validation and ultimately in vivo validation in mice.

Although Lee and authors applied their pipeline to a very specific tissue (i.e., lung) and senescence inducer (i.e., bleomycin-induced fibrotic lung injury), the approach could be broadly applied (Figure 1). The paper itself is an elegant case-study on the application of this pipeline to search for senolytics that may be effective in treating a specific disease, idiopathic pulmonary fibrosis (IPF), which has previously been associated with the accumulation of senescent cells in the lung (15, 16). As an albeit imperfect but reasonable surrogate for IPF, the authors treated INKBRITE mice with bleomycin (15, 17). Fourteen days following bleomycin administration, they sorted GFP^+^ and GFP^–^ fibroblasts (CD45^–^EpCAM^–^CD31^–^) from the fibrotic lungs. In order to directly compare the potency of candidate senolytic compounds in selectively killing senescent cells, the authors mixed the GFP^+^ and GFP^–^ cells in a 1:1 ratio and then performed a high-throughput drug screen using approximately 2,000 small molecules. The goal of this screen was to identify the most potent compounds that killed the GFP^+^ cells while sparing the GFP^–^ cells. Here again, they took advantage of the INKBRITE reporter and used image segmentation of GFP^+^ and GFP^–^ nuclei, identifying 37 compounds that reduced the percentage of GFP^+^ cells to less than three standard deviations of the percentage of GFP^+^ cells in control wells (corresponding to less than 20% GFP^+^ cells). Interestingly, senolytics previously identified using the traditional in vitro screening approach—dasatinib (4), quercetin (4), and fisetin (18) — also reduced the GFP signal, but not to below the 20% threshold, providing support for the hypothesis that this tissue-specific in vivo senescence approach may be superior to current in vitro approaches in identifying senolytics for specific disease states (15).

The subsequent steps generally followed the traditional approach of secondary validation with dose-response curves to determine the half-maximal inhibitory concentrations (IC_50_s) for reduction of percent GFP^+^ fibroblasts. Of the 32 compounds that underwent secondary screening, eight compounds had an IC_50_ below 2 μM, with most of these compounds being HSP90 and HDAC inhibitors. Before moving to in vivo validation, however, the authors performed an ex vivo screen using precision cut lung slices from bleomycin-injured INKBRITE mice and identified two HSP90 inhibitors (XL888 and ganetespib) as being the most promising compounds. XL888 was then shown to be an effective senolytic in vivo in mice following fibrotic lung injury and also in fibroblasts isolated from lungs of patients with IPF (15).

Lee et al. (15) is an important study for a number of reasons, and it also raises several unanswered questions. Notably, it describes a robust pipeline for isolating and performing high-throughput drug screening on cells where senescence was induced in vivo rather than under highly artificial in vitro conditions. Moreover, using the INKBRITE mice, this pipeline can be utilized for any tissue or senescence inducer, including aging. It also identifies that XL888 may have senolytic efficacy in IPF and opens an avenue for treating this devastating condition.

A limitation of using the INKBRITE mice in this pipeline, however, is that senolytic compounds are only identified for *p16^Ink4a^*-expressing cells, and not those in which senescence is driven principally by *p21^Cip1^*. Indeed, there is increasing evidence that in vivo, *p16^Ink4a^*- and *p21^Cip1^*-expressing cells are distinct and largely nonoverlapping (14, 19). As such, similar screens using *p21^Cip1^* reporter mice, or possibly double transgenic mice expressing both *p16^Ink4a^* and *p21^Cip1^* reporters, may be more comprehensive for identifying candidate senolytic compounds.

## Broad spectrum versus tissue- or disease-specific senolytics

Because the approach used by Lee et al. (15) focuses on specific tissues where senescence is induced in vivo, the question arises as to whether this method will inevitably lead to tissue- or disease-specific senolytics that may not be useful for treating multiple aging comorbidities across tissues. This idea may be troubling to supporters of the geroscience hypothesis, where the original vision was to have broad-spectrum senolytics that targeted fundamental aging mechanisms across tissues and could therefore ameliorate a range of age-related comorbidities simultaneously (e.g., dementia, diabetes, cardiovascular disease, osteoporosis, and others) (1). Pursuit of tissue-specific senolytics, while important for particular diseases, potentially undercuts this vision. Clearly, however, approaches attempting to achieve both goals are not mutually exclusive. Indeed, the pipeline developed by Lee et al. (15) could also be used to identify broad spectrum senolytics by evaluating a range of tissues in aged mice and discovering compounds with better activity across tissues than current senolytic compounds. The likely bet is that broad-spectrum (for attenuating aging across tissues) and tissue- and/or disease-specific (for treating particular diseases) senolytics will likely need to be developed in order to achieve the full potential of these promising compounds.

## Version 1 05/01/2024

Electronic publication
